# Quantification of Lappaconitine in Mouse Blood by UPLC-MS/MS and Its Application to a Pharmacokinetic Study

**DOI:** 10.1155/2019/6262105

**Published:** 2019-01-06

**Authors:** Fan Chen, Xiuwei Shen, Peng Huang, Huiyan Fu, Yue Jin, Congcong Wen

**Affiliations:** ^1^Ruian People's Hospital, The Third Affiliated Hospital of Wenzhou Medical University, Wenzhou 325000, China; ^2^Laboratory Animal Centre, Wenzhou Medical University, Wenzhou 325035, China

## Abstract

Lappaconitine is extracted from* Aconitum sinomontanum *Nakai, which belongs to the Ranunculaceae. Lappaconitine is as a diterpenoid alkaloid used as a nonaddictive analgesic. To assure the rational use of the drug, ultrahigh-pressure liquid chromatography tandem mass spectrometry (UPLC-MS/MS) was conducted to determine lappaconitine in mouse blood and its application to pharmacokinetics. In this study, khasianine was used as internet standard (IS). A UPLC BEH C18 column was used for chromatographic separation and the mobile phase consisted of acetonitrile and 10 mmol/L ammonium acetate (0.1% formic acid). The flow rate of was 0.4 mL/min. Quantitative detection was performed in a multiple reaction monitoring (MRM) mode using an electrospray ionization source in positive mode. Twenty-four mice were randomly divided into four groups, three of which received 2, 4, and 8 mg/kg lappaconitine by intragastric administration, while the other group received 1 mg/kg lappaconitine by intravenous administration. After 0.0833, 0.5, 1, 1.5, 2, 3, 4, and 8 h, blood samples were collected and acetonitrile was used for protein precipitation. A linear calibration relationship (R^2^ = 0.9979) in the range of 0.1-500 ng/mL in mouse blood indicated good results. The lower limit of quantitation was 0.1 ng/mL and the limit of detection was 0.04 ng/mL. The intra-day and inter-day precision were below 13% and 14%, respectively. The accuracy was 90.1-107.2%, and the recovery exceeded 81.1%. The matrix effect ranged between 102.1 and 108.8%. The absolute bioavailability of lappaconitine was 2.0%. UPLC-MS/MS achieved high sensitivity, speed, and selectivity. Methodological verification indicated this method as suitable for determination of lappaconitine in mouse blood.

## 1. Introduction

Lappaconitine (LA) has a varied usage history [1]. LA, which is also called aconitum alkaloid, is extracted from* Aconitum sinomontanum *Nakai. In the clinical context, LA is used as a nonaddictive analgesic [2]. Furthermore, LA also showed anesthesia, antipyretic, anti-inflammatory swelling, antitumor, and antiarrhythmia activities [3, 4]. Compared to aminopyrine, LA has a stronger neuroleptic ability. LA has similar sedative capability to meperidine and is neither teratogenic nor mutagenic. The pharmacological activity of LA is long lasting. As a type of aconitum, toxicity may occur even at small dosages [1, 5]. LA might induce ventricular arrhythmias, respiratory depression, and convulsions [6, 7]. Ameri reported that the structure of LA had strong pharmacological activity and low toxicity [1].

Several studies reported the determination of LA in mouse plasma or rabbit plasma [8, 9]. Wang et al. developed a highly sensitive and rapid method for the analysis of lappaconitine in mouse plasma using liquid chromatography coupled with mass spectrometry (LC-MS). The LLOQ was 2 ng/mL and the total runtime was 5 min [8]. Wang et al. also developed a highly sensitive and rapid method for the analysis of lappaconitine in rabbit plasma using LC-MS/MS. This method was linear over the concentration range of 13.125-1050.0 ng/mL, the lower limit of quantification was 13.125 ng/mL, and the total runtime was 7.5 min. Compared to the LC method, the UPLC-MS/MS method was more sensitive and rapid, and its high separating capacity was more suitable for complex compounds in biological samples [10, 11]. In this study, to establish a sensitive, stable, and accurate UPLC-MS/MS method for the determination of LA in mouse blood and its application to pharmacokinetics.

## 2. Experimental

### 2.1. Chemicals and Animal

**Figure 1:**
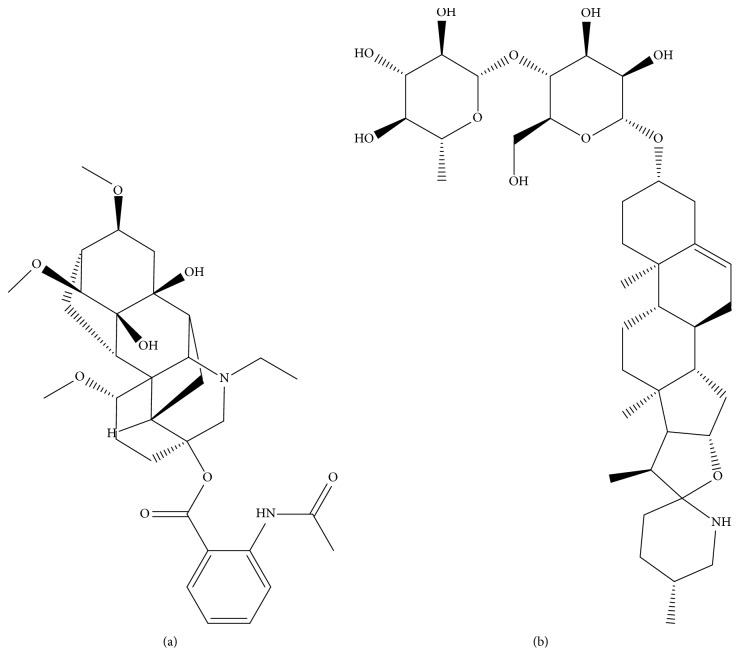
Chemical structures of LA (a) and IS (b).

LA (purity > 98%, Figure 1(a)) and IS (purity > 98%, Figure 1(b)) were purchased from the Chengdu Manchester Pharmaceutical Company. Acetonitrile and methanol (LC grade) were purchased from Merck. Ultra-water was made with a Millipore Milli-Q Purification system. Institute of Cancer Research (ICR) mice were provided by the Wenzhou Medical University.

### 2.2. Instrumentation and Conditions

UPLC-MS/MS was equipped with ACQUITY I-Class UPLC and XEVO TQS-micro Triple Quadrupole Mass Spectrometer.

UPLC BEH C18 (2.1 mm × 50 mm, 1.7 *μ*m) was used for chromatographic separation. The column temperature was set to 30°C at a flow rate of 0.4 mL/min. The mobile phase was comprised of acetonitrile and 10 mmol/L ammonium acetate (0.1% formic acid). The total run time was 4 min and mobile phase changed as follows: 0-0.2 min, acetonitrile was maintained at 10%; acetonitrile was increased from 10% to 80% in the period from 0.2 to 1.5 min; 80% acetonitrile was retained in 0.5 min. 2.0-2.5 min, acetonitrile was decreased to 10% and acetonitrile was maintained at 10% until 4.0 min.

**Figure 2:**
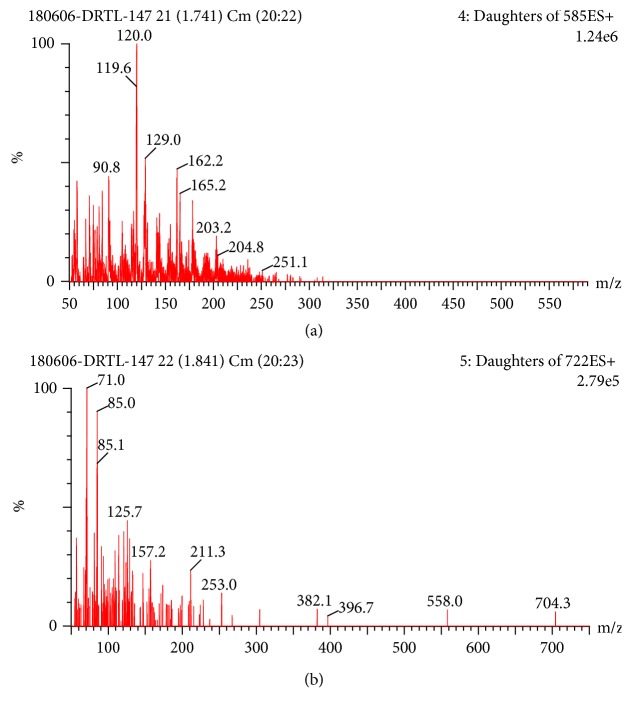
Mass spectra of LA (a) and IS (b).

Nitrogen was used as desolvated gas (800 L/h, 400°C). The capillary voltage was 2.0 kV and the ion source temperature was 150°C. MRM transitions of LA and IS were 585.3 → 119.9 and m/z 722.4 → 70.7 (Figure 2), respectively.

### 2.3. Standard and QC Preparation

Stock solution of LA (1.0 mg/mL) and IS (1.0 mg/mL) were prepared by methanol-water (v: v, 1/1). The series work solution of LA was prepared by methanol. The work solution of IS (20 ng/mL) was prepared by acetonitrile.

After adding appropriate work solution of LA to blank blood, the final calibration of LA in mouse blood was 0.1, 0.5, 2, 5, 20, 50, 200, and 500 ng/mL. Quality control (QC) (0.1, 0.4, 15, and 450 ng/mL) was prepared identical to calibration. All standards were used at 4°C.

### 2.4. Sample Preparation

The 100 *μ*L acetonitrile (including 20 ng/mL IS) was added into 20 *μ*L blood sample in a 1.5 mL centrifuge tube, then vortexed for 1.0 min, and centrifuged at 13,000 rpm for 10 min. The supernatant (80 *μ*L) was collected and 2 *μ*L was injected into UPLC-MS/MS for analysis.

### 2.5. Method Validation

The verification method was established in accordance with the US Food and Drug Administration (FDA) bioanalytical method validation guidelines. The verification included selectivity, matrix effects, linearity, precision, accuracy, recovery, and stability [12].

#### 2.5.1. Selectivity

Six lots of blank blood samples and a blank blood sample spiked with LA and IS were used for evaluation of selectivity.

#### 2.5.2. Linearity

In the concentration range of 0.1-500 ng/mL, a calibration curve was established by plotting the ratio of the analyte peak area to the IS peak area versus the analyte concentration.

#### 2.5.3. Precision and Accuracy

Precision and accuracy were evaluated by six repetitions of QC samples (0.1, 0.4, 15, and 450 ng/mL). Intra-day and inter-day precision were determined by measuring QC samples for three consecutive days. Precision is shown as the relative standard deviation (RSD). Intra-day and inter-day accuracy were determined by the ratio of the average of the measured concentration to the standard concentration.

#### 2.5.4. Recovery and Matrix Effects

To obtain the value of recovery, peak areas of QC samples (0.1, 0.4, 15, and 450 ng/mL) were compared to the corresponding concentration. Adding pre-blood samples to the standard (0.1, 0.4, 15, and 450 ng/mL) obtained peak A. Peak B was achieved by dilution sample with acetonitrile/0.1% formic acid (v/v=1:1). Matrix effects were assumed as the ratio of Peak A to Peak B.

#### 2.5.5. Stability

Stability was evaluated for four conditions: firstly, the stability in injection bottles; secondly, short-term stability (at room temperature for 2 h); thirdly, long-term stability (-20°C for 30 days); lastly, freeze-thaw stability (-20°C and room temperature for three times).

### 2.6. Pharmacokinetics

LA (1.0 mg/mL) was prepared with 0.01% HCl prior to animal experiment. Twenty-four mice were randomly divided into four groups. IV group received 1 mg/mL LA via intravenous administration; the remaining three groups received 2 mg/kg, 4 mg/kg, and 8 mg/kg of LA via intragastric administration. After 0.0833, 0.5, 1, 1.5, 2, 3, 4, and 8 h, 20 *μ*L blood sample of each group was collected into 1.5 mL tubes. All samples were stored at -20°C.

Area under blood concentration-time curve (AUC), mean residence time (MRT), blood clearance (CL), apparent volume of distribution (V), maximum blood concentration (Cmax), and half-life (t_1/2_) were calculated via DAS 2.0 in a noncompartmental model. Data are shown as means ± SD. Absolute bioavailability = AUC of intragastric administration / AUC of intravenous.

## 3. Result and Discussion

### 3.1. Method Optimization

The choice of positive and negative electrodes for electrospray (ESI) is often evaluated in methodology [13, 14]. LA is an alkaloid that is suitable for ESI positive mode.

The UPLC condition will be for the separation of IS and LA from endogenous interfering substance [15–17]. The chosen of column and mobile phase were responsible for chromatography separation. Acetonitrile-0.1% formic acid, acetonitrile-10 mmol/L ammonium acetate (0.1% formic acid), methanol-0.1% formic acid, and methanol-10 mmol/L ammonium acetate (0.1% formic acid) were used for the mobile phase. The results showed that acetonitrile-10 mmol/L ammonium acetate (0.1% formic acid) obtained the most satisfactory chromatographic peak shape and retention time.

Compared to LC-MS/MS detection, the UPLC-MS/MS was faster and more sensitive [8, 9]. The new UPLC-MS/MS method was only used for 4 min, and LLOQ was 0.1 ng/mL.

### 3.2. Method Validation

**Figure 3:**
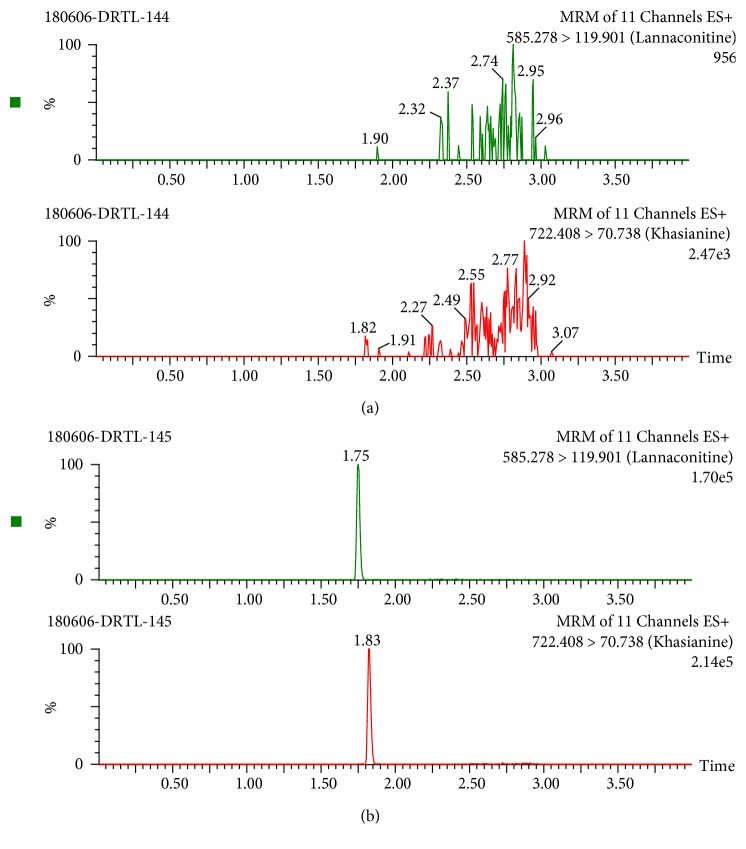
UPLC-MS/MS LA and IS in mouse blood: (a) blank blood sample; (b) blank blood sample spiked with 10 ng/mL LA and 20 ng/mL IS.

Figure 3 shows that the retention times of LA and IS were 1.75 and 1.83 min. Figure 3 shows that there were no obvious impurities or interferes.

Calibration of blood sample was Y = 0.0017C + 0.0028, R^2^ = 0.9979, where Y represents the ratio of peak area to IS peak area and C represents the blood concentration of LA. LLOQ was 0.1 ng/mL with a signal noise ratio of 8. LOD was 0.04 ng/mL with a signal noise ratio of 3.

The RSDs of inter- and intra-precision were less than 13% and 14%, respectively. Accuracy was in a range from 90.1% to 107.2%. Recovery was better than 81.1%. The matrix effect was between 102.1% and 108.8%. The data was listed in Table 1. These results showed that the method meet a criterion of the pharmacokinetics.

The stability of this method showed variation of LA within ±15% in four conditions.

### 3.3. Pharmacokinetics

**Figure 4:**
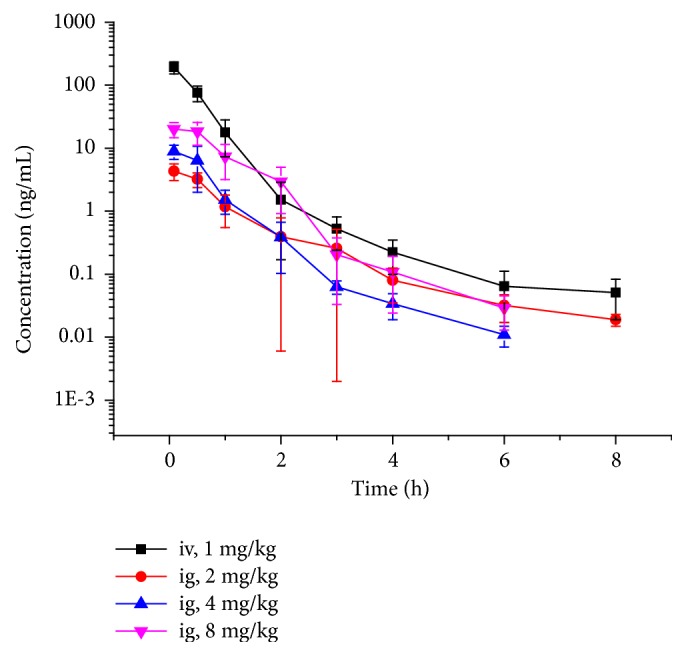
Mean blood concentration versus time after intravenous (1 mg/kg) or intragastric (2, 4, or 8 mg/kg) administration of LA.

LA is a nonnarcotic central nervous system analgesic with strong action. The analgesic activity of LA exceeds that of both indometacin and acetylsalicylic acid; however, it is about 2 to 5 times less than that of morphine [8]. The concentration of LA in mice detected by UPLC-MS/MS is shown in Figure 4. Furthermore, the main pharmacokinetic parameters were calculated by DAS 2.0 and are listed in Table 2. Table 2 shows that the AUC of the intravenous group was higher than that of all intragastric groups. The absolute bioavailability was only 2.0%, and no other study reported the bioavailability of LA. This result might be due to poor oral administration. The AUC of intragastric administration at 2, 4, and 8 mg/kg are not proportional, which may be due to the low bioavailability.

The concentration versus time curve during 24 h was fitted using the bimodal model of DAS software (version 1.0) and the results showed that the elimination process of LA in the body of rabbits was similar to the one-compartment model [9]. After single intravenous injections of LA at 1.0, 2.0, and 4.0 mg/kg, the elimination half-lives (t_1/2_) were 0.47, 0.48, and 0.49 h, and the areas under the curve (AUC_0−t_) were 55.5, 110.5, and 402.9 ng/mL·h, respectively [8]. The t_1/2_ and AUC_0−t_ were 1.24 ± 0.52 and 109.8 ± 20.7 (Table 2). The different results may be due to differences between plasma and blood, while blood was used in this study for the determination of the LA concentration, and the AUC_0−t_ was lower than that reported in literature by Wang et al. [8].

## 4. Conclusion

A sensitivity, fast effect, and high selectivity UPLC-MS/MS method was developed and validated; with LLOQ (0.1 ng/mL), only 20 uL blood sample was required. This method was successfully applied to the pharmacokinetics of LA in mice

## Figures and Tables

**Table 1:** Precision, accuracy, matrix effect, and recovery for LA of QC sample in mouse blood (n = 6).

Concentration (ng/mL)	Accuracy (%)		Precision (RSD%)		Matrix Effect (%)	Recovery (%)
	Intra-day	Inter-day	Intra-day	Inter-day		
0.1	94.1	90.1	12.0	13.9	107.5 ± 6.2	81.4 ± 5.2
0.4	104.4	107.2	6.4	11.9	102.1 ± 6.7	87.0 ± 4.8
15	106.5	105.3	8.7	7.7	108.8 ± 7.7	81.1 ± 4.7
450	99.8	102.7	8.4	7.3	102.5 ± 2.6	85.0 ± 2.2

**Table 2:** Main pharmacokinetic parameters of LA in mouse blood after intravenous (iv; 1 mg/kg) or intragastric (ig; 2, 4, or 8 mg/kg) administration of LA.

Parameters	Unit	iv (1 mg/kg)	ig (2 mg/kg)	ig (4 mg/kg)	ig (8 mg/kg)
AUC(0-t)	ng/mL*∗*h	109.8±20.7	4.3±1.2	6.8±3.0	22.3±6.6
AUC(0-∞)	ng/mL *∗*h	109.9±20.6	4.3±1.2	6.8±3.0	22.4±6.6
MRT(0-t)	h	0.38±0.10	0.93±0.29	0.58±0.08	0.76±0.17
MRT(0-∞)	h	0.38±0.10	1.01±0.28	0.59±0.08	0.77 ±0.16
t1/2z	h	1.24±0.52	1.45±0.40	0.61±0.17	0.77 ±0.24
CLz/F	L/h/kg	9.4±2.0	497.7±169.1	681.4±274.4	395.8±165.3
Vz/F	L/kg	17.4±10.0	1063.9±485.2	611.3±365.3	438.2 ±226.9
Cmax	ng/mL	193.1±42.1	4.5±1.2	9.4±2.3	23.4 ±3.8
Bioavailability			2.0%	1.5%	2.5%

## Data Availability

The data used to support the findings of this study are available from the corresponding author upon request.
